# Filamentous Thermosensitive Mutant Z: An Appealing Target for Emerging Pathogens and a Trek on Its Natural Inhibitors

**DOI:** 10.3390/biology11050624

**Published:** 2022-04-20

**Authors:** Manisha Gurnani, Abhishek Chauhan, Anuj Ranjan, Hardeep Singh Tuli, Mustfa F. Alkhanani, Shafiul Haque, Kuldeep Dhama, Rup Lal, Tanu Jindal

**Affiliations:** 1 Amity Institute of Environmental Science, Amity University, Noida 201301, India; manishagurnani10@gmail.com; 2 Amity Institute of Environmental Toxicology, Safety and Management, Amity University, Noida 201303, India; tjindal@amity.edu; 3 Academy of Biology and Biotechnology, Southern Federal University, 344006 Rostov-on-Don, Russia; 4 Department of Biotechnology, Maharishi Markandeshwar (Deemed to be University), Ambala 133207, India; hardeep.biotech@gmail.com; 5 Emergency Service Department, College of Applied Sciences, AlMaarefa University, Riyadh 11597, Saudi Arabia; mkhanani@mcst.edu.sa; 6 Research and Scientific Studies Unit, College of Nursing and Allied Health Sciences, Jazan University, Jazan 45142, Saudi Arabia; shafiul.haque@hotmail.com; 7 Faculty of Medicine, Görükle Campus, Bursa Uludağ University, Nilüfer, Bursa 16059, Turkey; 8 Division of Pathology, ICAR—Indian Veterinary Research Institute, Bareilly 243122, India; kdhama@rediffmail.com; 9 Department of Zoology, University of Delhi, Delhi 110021, India; ruplal@gmail.com

**Keywords:** antimicrobials, cytokinesis, divisome, drug discovery, MRSA, VRE, Fts-Z assembly, microtubules, cell division, anti-bacterial drugs

## Abstract

**Simple Summary:**

Antimicrobial resistance (AMR) is a pressing issue worldwide that must be addressed swiftly. It is driven by spontaneous evolution, bacterial mutation, and the dissemination of resistant genes via horizontal gene transfer. Researchers are working on many novel targets, which can become a pathway to inhibit harmful bacteria. Filamentous Thermosensitive mutant-Z (Fts-Z) is one such bacterial target that has gained popularity amongst scientists due to its conserved nature in bacteria and absence in eukaryotes. The aim of this work was to review the Fts-Z mechanism of action along with current studies on natural inhibitors for Fts-Z.

**Abstract:**

Antibiotic resistance is a major emerging issue in the health care sector, as highlighted by the WHO. Filamentous Thermosensitive mutant Z (Fts-Z) is gaining significant attention in the scientific community as a potential anti-bacterial target for fighting antibiotic resistance among several pathogenic bacteria. The Fts-Z plays a key role in bacterial cell division by allowing Z ring formation. Several in vitro and in silico experiments have demonstrated that inhibition of Fts-Z can lead to filamentous growth of the cells, and finally, cell death occurs. Many natural compounds that have successfully inhibited Fts-Z are also studied. This review article intended to highlight the structural–functional aspect of Fts-Z that leads to Z-ring formation and its contribution to the biochemistry and physiology of cells. The current trend of natural inhibitors of Fts-Z protein is also covered.

## 1. Introduction

The story of antibiotics began in the 1930s with the discovery of Penicillin, the first antibiotic, by Alexander Fleming. The next 40 years were a “golden era”, and even the current antibiotics are the outcome of research work done during that time [1]. In that phase, the discovery of antibiotics was considered to be a sign of victory over bacterial infections, but bacteria are developing resistance to several conventional antibiotics, and this is posing a serious threat to the health care sector. 

Our knowledge about microbes and their infection-causing tendencies has increased exponentially due to the extensive development of techniques for their study throughout the 20th century. This, in turn, has boosted our ability to prevent and treat infections and achieve better public health. However, exploring the depths of infection-causing pathways is still is a mystery that we need to solve [2].

The WHO has clearly emphasized the concern about antimicrobial resistance (AMR) that is brought about by the misuse of antibiotics and deliberate consumption through poultry and meat [3]. A WHO report released in February 2017 emphasized making improved antibiotics for some pathogens a priority and categorized them as ESKAPE pathogens (*E. faecium*, *S. aureus*, *K. pneumoniae*, *A. baumannii*, *P. aeruginosa*, *Enterobacter*) [4,5,6]. These pathogens are WHO AMR priority pathogens as they have developed resistance against antibiotics such as β lactams [7,8,9], fluroquinolones [10,11,12], macrolides [13,14], tetracyclines [15,16], and even last line defense antibiotics like glycopeptides [17,18] and carbapenems [19,20,21]. They have also developed resistance against the clinically unfavorable polymyxins through mutations in genetic material and/or through assessing the MGEs, i.e., mobile genetic elements [22]. In the worst cases, AMR leads to the development of “Superbugs”, which develop multi-drug resistance (MDR) in bacteria [23,24]. New Delhi metallobeta-lactamase-1 (NDM-1) is another case of antibiotic resistance where, to date, colistin and tigecycline are the only effective antibiotics against these superbugs [25]. 

Antibiotic-resistant bacteria have been classified into three classes, namely:

(I) Methicillin-resistant *Staphylococcus aureus,* popularly known as (MRSA), which accounts for ~12,000 [7] deaths per year and a health care cost of ~USD 5 billion per year [26]. Following MRSA, Vancomycin-resistant *S. aureus* (VRSA) is another challenge to tackle and has become a new problem in hospitals [27].
(II) Pan drug-resistant and Multidrug-resistant, popularly known as PDR and MDR respectively, in Gram-negative bacterial strains of *E. coli*, *K. pneumoniae*, *A. baumannii*, *P. aeruginosa*, pose the threat of untreatable infections [28]. The avenues for finding novel antibiotics for these are limited due to their outer membrane, which prevents the entry of some antibiotics, and efflux pumps expel many of the remainders [29,30,31].
(III) Multidrug-resistant and extensively drug-resistant strains of *M. tuberculosis*, also called MDR-TB and XDR-TB, pose a threat to the developing world specifically. The treatment requires a 2-year course of antibiotics and has serious side effects for patients. XDR-TB is more difficult to cure and often fatal [32,33].

Many studies have been conducted in this area leading to the identification of novel antibiotics, identification of new targets, and management of AMR diseases. The research community is constantly working on the above strategy to explore the new unexplored boundaries. New pharmacophores showing good affinity against any key bacterial receptors are checked for future avenues, and their derivatives are also being worked upon [34]. The study of novel antibiotic compounds and new targets is the only way forward in health care research. In this context, in silico discoveries of novel compounds have become most popular. Currently, AMR has made it imperative to work on both ends of drug discovery; therefore, the ligand-based approach and the receptor-based approach must go hand in hand. The rigorous exploration for novel bacterial targets indicates bacterial divisome machinery to be an interesting receptor against antimicrobial activity [35]. 

## 2. Antibiotics and Bacterial Divisome Proteins as Emerging Therapeutic Targets

**Figure 1:**
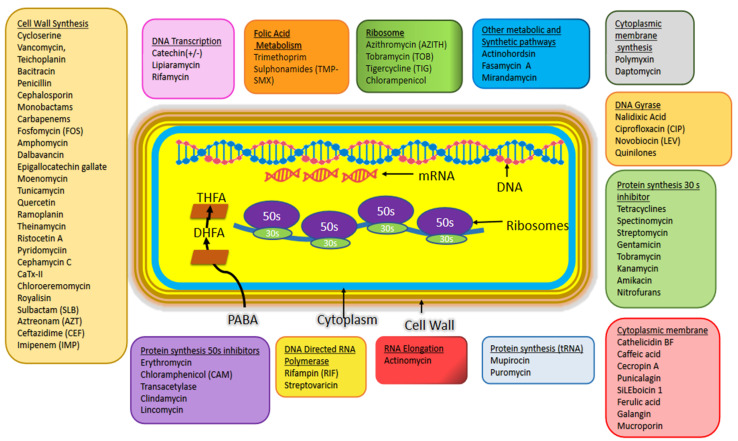
Antibiotics and their target sites in bacterial cells. This figure is adapted from: Bbosa et al., 2014 and Mendes et al., 2019 [43,44].

The emerging antimicrobial drug resistant in pathogens have made it mandatory to search for the new scaffolds both for drugs and targets on priority. From the past studies it is evident that the existing set of antibiotics is a combination of small molecules and through various synthetic techniques their effectivity has been increased by chemists and druggists [36,37,38,39,40,41]. Figure 1 shows the known antibiotics and their target sites in pathogen causing bacteria. Potential targets have been discovered with the help of genomic data of these pathogens [42]. Many lead molecules are under preclinical trials for one or the other targets [43]. 

Bacterial cell division is being considered as the new antibiotic target and efforts are being made to identify compounds that either directly interact with the major divisome machinery or affect the structural integrity of Fts-Z by inhibiting its activity [35]. A few other targets from divisome machinery on which studies have already been done are Fts-A [45], Fts-BLQ, Fts-N [46], Fts-K [47], Fts-Q [48] Fts-W [49]. Some of these studies include divisome proteins to be potent targets while others aim to understand the roles of these in division to find new targets against pathogens. The functions of some of the essential cell division proteins has been summarized in Table 1. 

## 3. Fts-Z: An Appealing Antibacterial Target

Generally, bacteria undergo binary fission wherein, the divisome machinery coordinates the entire process with varying constituents across the species. In Gram negative bacteria the cell division process occurs in two phases: Early Divisome where the assembly of the Z ring occurs and Late Divisome where remodeling of Peptidoglycan and Septation occurs. Formation of the division site is the first step followed by elongation, septum formation, then chromosome is divided and lastly at the division site two daughter nuclei are formed [50,53,54]. The divisome complex comprises of more than 30 genes (including the important ones—Fts A, Fts B, Fts I, Fts K, Fts L, Fts N, Fts Q, Fts W, Fts Z and Zip A) of which Fts Z, a 40 kDa septal ring progenitor protein with 383 residues, is responsible for proto ring formation. It is after this gene only that the splitting initiates [54,55,56]. For this reason, it is extensively explored. It has been named so because its mutant causes filamentation in *E. coli* cells at extreme temperatures, thus called “filamentous temperature sensitive” [57,58,59,60,61,62]. It forms a dais for assembly and is the prime energy source for cell wall constriction [63,64,65,66,67,68,69]. Fts-Z forms ring like constriction ‘the Z-ring’ (which is usually at the midpoint of the rod lying perpendicular to the long cell axis in *E. coli* and *B. subtilis*), and further downstream recruitment of ~13 other proteins take place which guides the location, synthesis, and shape of division septum. Many molecular mechanisms such as Zap and Min proteins, work together to govern Z ring placement [70]. Tethering of the membrane is mediated by Fts-A (along with Zip A in Gram-positive and Sep F in Gram-negative microbes). This complex of Fts-Z along with Fts-A (Sep F/Zip A) is a ‘proto-ring’. Fts-Z driven constriction has been defined by two well-known models: the sliding model and the bending model. The first model is dependent on the treadmilling of Fts-Z monomers causing the sliding of filaments against each other and the tightening of the ring occurs by overlapping filaments. The bending model is based on conformations change in protomers during GTP hydrolysis which induces a curvature in filaments. This happens only when Fts-Z is attached to the membrane so that a force is exerted on the membrane allowing invagination when peptidoglycan remodeling enzymes are present [47,71]. 

**Figure 2:**
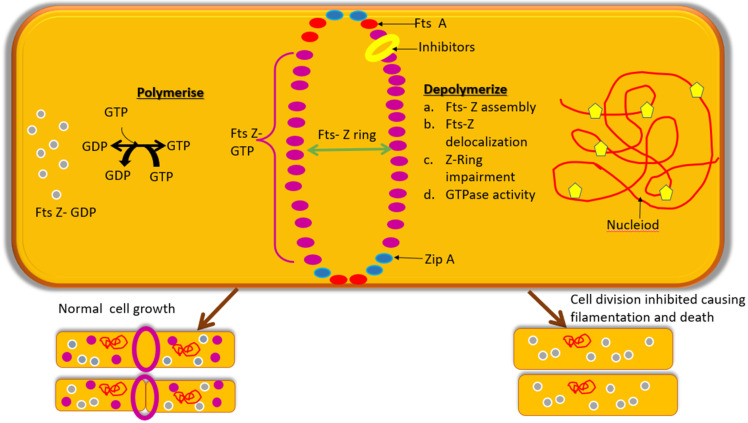
Polymerization and Depolymerization of Fts-Z ring in the presence and absence of inhibitors (yellow pentagon).

Due to its conserved nature amongst nearly all prokaryotes, and absence in the eukaryotes, it is being considered to be an appealing target for forming antibiotics and thus the molecules which can inhibit its activity will eventually disrupt the bacterial viability of pathogens. Figure 2 shows how polymerization occurs in normal Fts-Z ring formation and also the way Fts-Z molecules depolymerize in the presence of inhibitors. The mechanism of action of Fts-Z inhibitors can be: (a) through Fts-Z assembly inhibition [72,73,74]; (b) through entire Z-ring inhibition as done by berberine [48], cinnamaldehyde [75]; (c) through stimulating or discontinuing Fts-Z polymerization to disturb cytokinesis as done by taxanes [76,77], Mci-Z [78] etc.; (d) elevating or hindering GTPase activity to obstruct cytokinesis as done by curcumin [53], benzimidazoles; Fts-Z delocalization through point foci formation as done by certain synthetic compounds [79]. These all will cause the cell to elongate into a filamentous structure and eventually will result in cell death [80]. 

### Structural and Functional Aspect of Fts-Z

**Figure 3:**
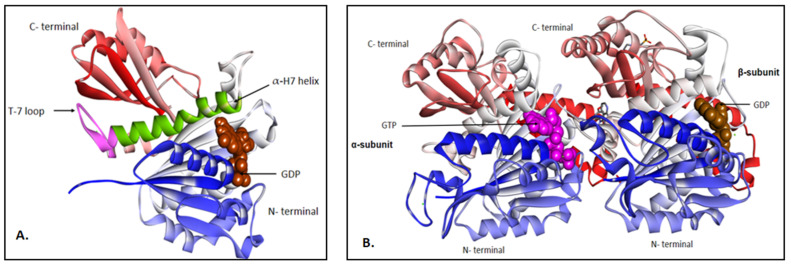
(**A**) Fts-Z protein from *S. aureus* (PDB ID—6RVQ) in ribbons bound to GDP (in brown CPK). The T7 loop is in pink and α-H7 helix is in green color. After the T7 loop starts the H8 helix. (**B**) Tubulin protein from *Homo sapiens* (PDB ID—6BR1) in ribbons showing alpha and beta subunits bound to GTP (in pink CPK) and GDP (in brown CPK) respectively. Note: Blue colored portion is the N-terminal and the green portion is the C-terminal. The figure was visualized in BIOVIA Discovery studio visualizer v21.1.

The Fts-Z protein is broadly characterized into 5 portions: 2 conserved globular subdomains, N-terminal starting from residue 13 to residue 178 and C-terminal starting from residue 209 to 314, connected to each other by a central helix (α-H7 starting from residue 179 to 202) and a synergy loop (T7) which connects α-H7 and H8., (Figure 3A) [81]. The N-terminal subunit and the C-terminal linker which is quite unstructured and least conserved, the C terminal tail responsible for interacting/contacting between Fts-Z and other auxiliary proteins for the formation of protofilaments of Z-ring and the C-terminal variable which makes lateral contacts if the modulatory proteins are not present at the site [82]. N-terminal has nucleotide binding motif with parallel beta sheets connected to α helices called “Rossmann fold”. Thus, the binding cavity of this protein is formed by the conjugation point between the two monomer units and GTP hydrolyses rely on their assembly, which is further dependent on the binding of Mg^++^ ion and monovalent cations [57].

Certain studies indicate the structural homology of this crucial protein with human tubulin protein, sharing a common ancestry although significant differences between amino-acid sequences were reported. Both the proteins tether and hydrolyze GTP and polymerize into a GTP dependent fashion which is another point where biochemical similarity is revealed [83,84]. Sequence motifs of Fts-Z like GGGTGTG, are also monograms of alpha-, beta- and gamma- tubulins and are connected to GTP binding ability. Another important motif, G-box, a seven amino acid sequence (SAG)GGTG(SAT)G was also found to be conserved in the tubulin family from different species [59]. Studies of the archaeal group gave indications that Fts-Z might have evolved to tubulin [60]. The variation in the structure was reported at the hydrophobic cleft which is at H7 where the N terminus is connected to the C terminus [85,86]. This hydrophobic cleft is absent in eukaryotic protein which is shown in Figure 3B.

## 4. Fts-Z Inhibition: A Strategy to Combat AMR

Fts-Z is being explored as potent target for inhibiting emerging microbes due to its central role in Z-ring formation and conserved nature in nearly all bacterial species and absence in higher eukaryotes. Its absence in bacteria induces filamentation and cell death occurs [85]. 

**Figure 4:**
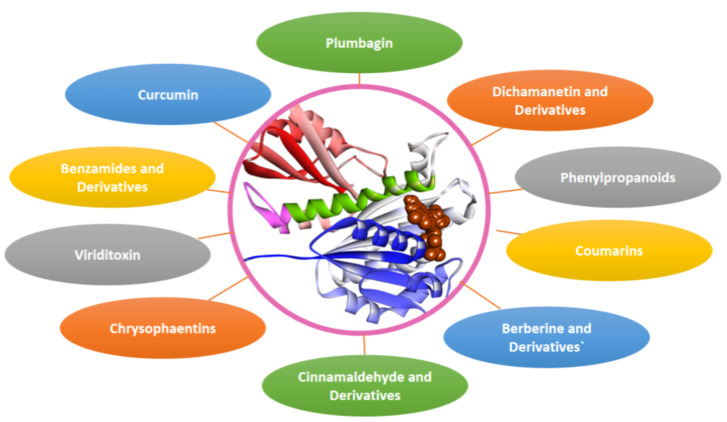
Fts-Z natural inhibitors.

Research work is being carried out to screen phytocompounds and other natural compounds using in-vitro and in-silico techniques. Some of the natural compounds such as berberine [87], curcumin [88], cinnamaldehyde [89], plumbagin [90], viriditoxin [91], dichamanetins [92], coumarins [93], Chrysophaentins [94] and some phenylpropanoids [95] and some have recently been investigated for Fts-Z inhibitors and antimicrobial properties. A few potential inhibitors of Fts-Z are discussed below (Figure 4). 

### 4.1. Berberine and Derivatives

**Figure 5:**
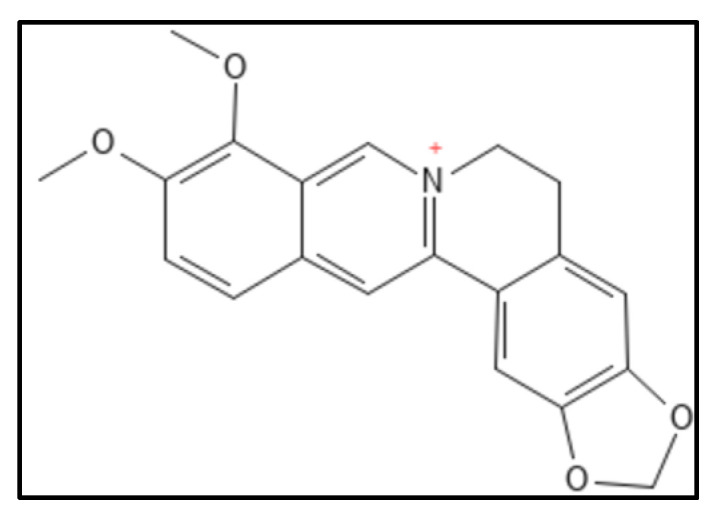
Berberine structure; Mol. Formula—C_20_H_18_NO_4_^+^; Mol. Wt.—336.4; Experimental details—berberine-dependent Fts-Z assembly observed (IC_50_ = 10.0 ± 2.5 µM); Fts-Z GTPase activity observed (IC_50_ = 16.01 ± 5.0 µM), ITC results—dissociation constant (K_D_ = 0.023) entropy driven [61]. Source: PubChem-CID 2353.

**Figure 6:**
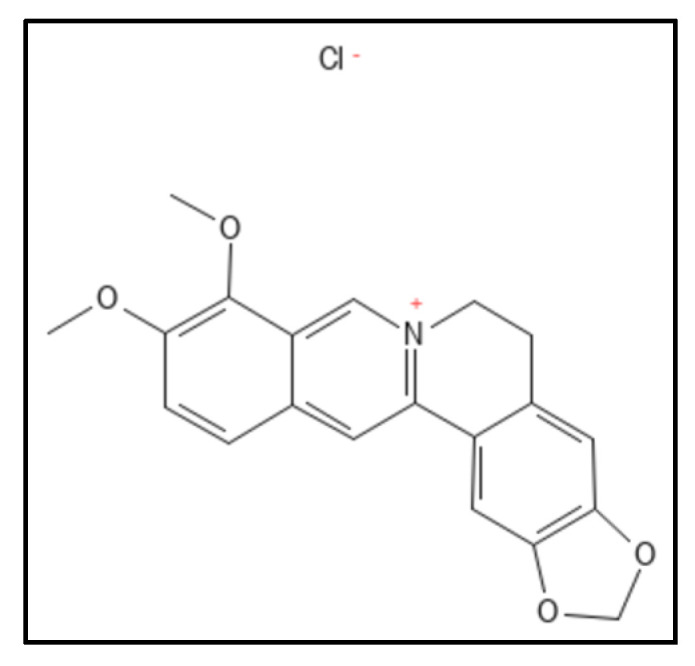
Berberine chloride derivative; Mol. Formula—C_20_H_18_ClNO_4_; Mol. Wt.—371.8; Experimental details—Inhibited MRSA ATCC 29247 (MIC = 2 µg mL^−1^), ATCC 700221 (MIC = 4 µg mL^−1^), *E. coli* (MIC = 32 µg mL^−1^), *K. pneumoniae* (MIC = 64 µg mL^−1^); showed GTPase (IC_50_ between 37.8 and 63.7 µM). In-silico studies done on PDB-4DXD (interacting residues were Ile197, Leu200, Val203, Leu209, Met226, Leu261, Val297 and Ile311) [61]. Source: PubChem-CID 12456.

Berberine (IUPAC: 16,17-dimethoxy-5,7-dioxa-13-azoniapentacy-clo [11.8.0.0^2,10^.0^4,8^.0^15,20^] henicosa-1(13),2,4(8),9,14,16,18,20-octaene), a benzyl isoquinoline alkaloid (Figure 5) from the Berberis plant, show a mild antibacterial activity and Fts- Z inhibition. Sun et al. in their work, studied the active site in *S. aureus* for Fts-Z (PDB ID—4DXD) binding through in-silico technique and the found interdomain region participating actively in the docking comparable to that of PC190723 [61]. The planar structure of the compound is best suited for interaction with the C-terminus beta sheet of Fts-Z protein. Further they designed and synthesized 9-phenoxyalkyl substituted derivatives of berberine (Figure 6) and found a compound that has a high potent activity which was further verified through in-vitro antimicrobial susceptibility assay and GTPase activity assay. Dasgupta and colleagues, a few years earlier had reported the inhibitory activity of berberine through In-vitro isothermal colorimetry (ITC) and STD NMR spectroscopy [75]. The studies indicated the overlap of the binding site of berberine with the GTP binding pocket in the Fts-Z protein. Berberine also had low toxicity rates and some other side effects like that of bilirubin -induced brain damage causing jaundice in infants and expecting mothers [87,96,97]. 

### 4.2. Sanguinarine

**Figure 7:**
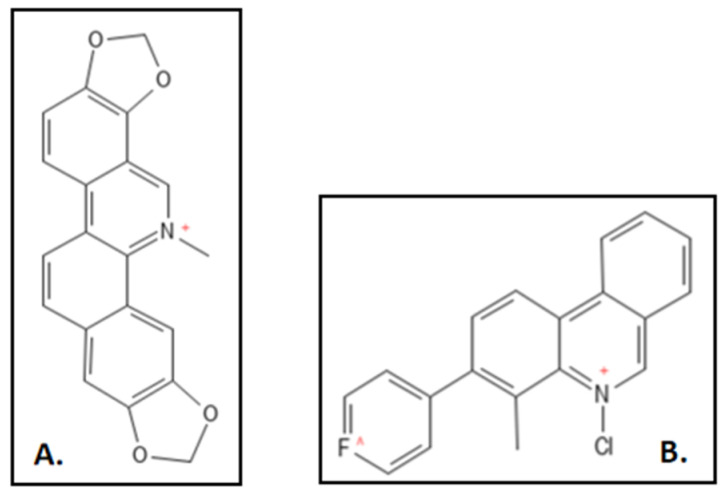
(**A**) Sanguinarine; Mol. Formula—C_20_H_14_NO_4_^+^; Mol. Wt.—332.3; Experimental details—Dissociation constant (K_D_) = 18–20 µM; IC_50_ = 3 ± 1 µM for *B. subtilis* 168, 14 ± 2.3 µM for *E. coli* BL21 [89]. (**B**) 5-methyl-2-phenylphenanthridium derivative of Sanguinarine [97]. Source: PubChem-CID 5154; PMID-29657101.

Sanguinarine (IUPAC: 24-methyl-5,7,18,20-tetraoxa-24-azoniahexacy-clo [11.11.0.0^2,10^.0^4,8^.0^14,22^.0^17,21^] tetracosa-1(24),2,4(8),9,11,13,15,17(21),22-nonaene) is a benzophenanthridine alkaloid from rhizome of *Sanguinaria canadensis* is found to inhibit cytokinesis in bacteria. It inhibits the *E. coli* Fts-Z assembly by perturbing the Z ring and was also found to inhibit *B. subtilis* cell growth without affecting nucleoid segregation at an IC_50_ of 3µM. This interaction was investigated through in-vitro size exclusion chromatography and fluoroscent probing. Mutational studies were also conducted by the group (Figure 7A) [90]. A representative compound 21 had 2 fold higher GTPase activity as compared to sanguinarine for which a patent was also filed [98]. Liu and coworkers designed and synthesized sequence of novel 5-methyl-2-phenylphenanthridium (Figure 7B) derivatives by simplifying the skeleton of sanguinarine. These had an exceptionally elevated anti-bacterial activity against an array of 10 antibiotic sensitive and resistant strains. MIC values for *S. aureus* ATCC25923, *S. epidermis*, *S. pyogenes PS* and *PR*, *B. subtilis* ATCC9372, *B. pumilus* ATCC63202 were ranged from 0.06 to 8 and 0.25–16 µg mL^−1^ respectively and for *E. coli* ATCC 25922 and *P. aeruginosa* ATCC27853 it was more than 64 µg mL^−1^ [89]. 

### 4.3. Cinnamaldehyde and Derivatives

**Figure 8:**
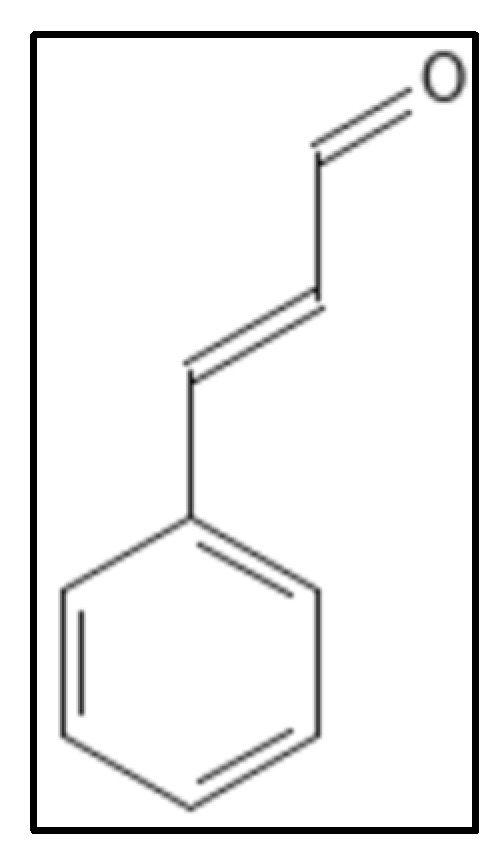
Cinnamaldehyde structure. Mol. Formula—C_9_H_8_O; Mol. Wt.—132.16; Experimental details—inhibits *E. coli* (MIC = 1000 mgL^−1^), *B. subtilis* (MIC = 500 mgL^−1^), MSRA (MIC = 250 mgL^−1^), GTPase inhibition (IC_50_ = 5.81 ± 2.2 µM). In-silico docking (PDB ID—1FSZ), interacting residue were V208, R202, N263, G295, S297 [89]. Source PubChem: CID 637511.

Cinnamaldehyde (IUPAC: (E)-3-phenylprop-2-enal), (Figure 8) a plant derived product from spices (stem bark of *Cinnamomum cassia*) has shown an array of potential medicinal properties. It has been reported to possess inhibitory activity against yeasts, filamentous molds and many bacteria through various pathways including inhibition of cell wall biosynthesis, changing of membrane structure and integrity and inhibiting GTPase activity [63]. Domadia and coworkers reported in-vitro, in-silico and in-vivo activity of cinnamaldehyde to perturb Z ring morphology and GTP hydrolysis with an affinity of 1.0 ± 0.2 µ/M [76]. Molecular modeling results were in concordance with STD-NMR results showing binding at the T7 loop in the C-terminal region. The results of MSA (multiple sequence alignment) also showed some of the residues- R202, V208, N263, G295, N263 to be highly conserved among Fts-Z. Xin and coworkers worked on derivatives of cinnamaldehyde where they found that substitution in the benzene ring at ortho or para position of cinnamaldehyde, by small groups increased the activity of compounds so synthesized against different microbes. They further stated that 2-methyl benzimidazoyl moiety was had a better efficiency against all strains, they tested and gave three compounds (marked as 3, 8, 10 in [94]) which had MIC of 4 µg mL^−1^ in two compounds and 10 µg mL^−1^ against *S. aureus* (ATCC 25923). Two compounds (marked as 4, 10 in [64]) gave MIC (4 µg mL^−1^)values 32 times better than the reference drugs used. 

In 2015, another group of researchers took this study one step further by synthesizing cinnamaldehyde derivatives and reported comparable activity of them wherein some possessed cell division inhibition properties in *S. aureus* between 0.25–4 µg mL^−1^. They found good activity when 2-methylbenzimidazolyl substitution was at the first position and 2,4-dichlorophenyl was at the third position. Polymerization inhibition and GTPase activity of *S. aureus* Fts-Z were shown in dose-dependent manner through biological assays of compounds [94].

### 4.4. Chrysophaentins 

**Figure 9:**
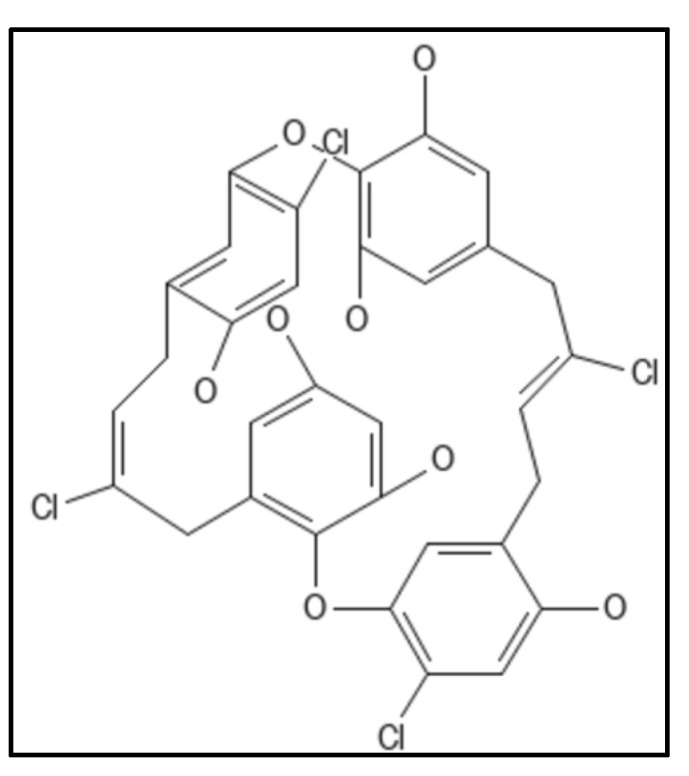
Chrysophaentin A Structure. Mol. Formula—C_32_H_24_Cl_4_O_8_; Mol. Wt.—678.3; Experimental details—GTPase activity (IC_50_ = 6.7 ± 1.7 µg mL^−1^); MIC_50_ = 1.8 ± 0.6 µg mL^−1^, 1.5 ± 0.7 µg mL^−1^, and 1.3 ± 0.4 µg mL^−1^ against SA, MRSA, and multi drug resistant SA (MDR-SA), respectively; and 3.8 ± 1.9 µg mL^−1^ and 2.9 ± 0.8 µg mL^−1^ toward *E. faecium* and VREF; In-silico docking on homology modelled E.coli, placed compound in GTP binding site (residues Gly20, Asn24, Asn 43, Ala 70, Thr 108, Arg 142 formed H-bond) [93]. Source: PubChem-CID 46872004.

**Figure 10:**
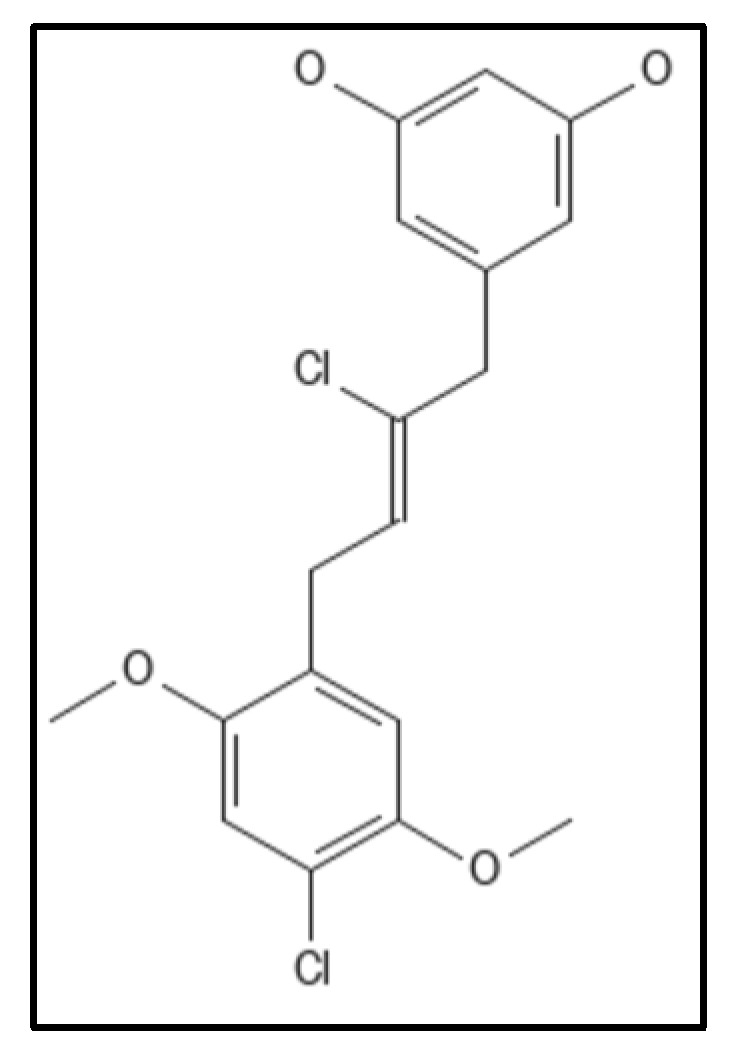
Hemichrysophaentin Structure; Mol. Formula—C_18_H_18_Cl_2_O_4_; Mol. Wt.—369.2 [93]. Source: PubChem-60164930.

Chrysophaentins are group of anti-infectives obtained from marine sources, chrysophyte alga, *Chryosphaeum taylori*, including 8 phytochemicals (A-H), showed the inhibitory activity for MRSA, VREF (vancomycin-resistant *E. faecium*) and *S. aureus* [93]. Of these, Chrysophaentin A (IUPAC: (9E,25E)-4,10,20,26-tetrachloro-2,18-dioxapentacy-clo [2 6.2.2.13,7.119,23.012,17]tetratriaconta-1(30),3,5,7(34),9,12(17),13,15,19,21,23(33),25,28,31-tetradecaene-6,14,16,22,30,31-hexol), (Figure 9) showed the most potent antibacterial activity and also inhibited GTPase activity in *E. coli* which was attributed to the presence of hydroxyl group. Also, Chrysophaentins A had 12-fold higher MIC_50_ as compared to Chrysophaentins D, due to the presence of chlorine at chains A and C. Another compound hemi-Chrysophaentins(Figure 10) was synthesized and reported to have a reaction system of Chrysophaentins A [67].

### 4.5. Coumarins

**Figure 11:**
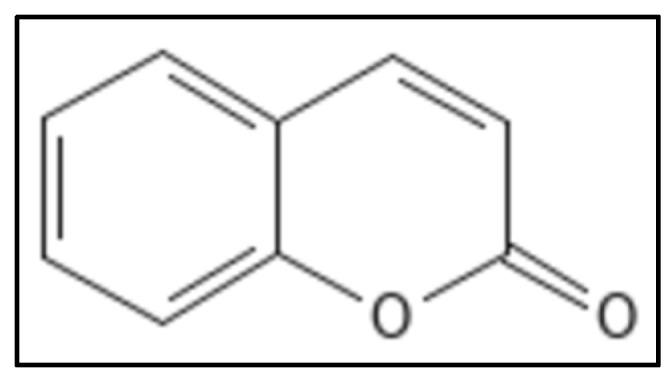
Coumarin Structure. Mol. Formula—C_9_H_6_O_2_; Mol. Wt.—146.14; GTPase activity (IC_50_ = 212 ± 4.12 µM); Fts-Z polymerization (IC_50_ = 200 ± 4.2 µM) [58]. Source: PubChem-323.

Coumarins (IUPAC: chromen-2-one), (Figure 11) are 1,2-benzopyrone derivatives were derived from different plants with a proven Fts-Z inhibition activity. They have a 2H-chromen-2-one composed of lactone and aromatic ring, along with 2 oxygen atoms forming bonds with enzyme residues and thus are responsible for pharmacological properties. The aromatic ring also plays role in destabilizing enzymes by forming hydrophobic bonds [69]. Ammoresinol, anthogenol, ostruthin, novobiocin, chartreusin and coumermycin are some of the coumarins which also show anti-bacterial activity against many Gram-negative and Gram-positive bacteria [99]. A study suggested that coumarins had potent activity against *M. tuberculosis* (H37Rv) by inhibiting GTPase activity and polymerization of Fts-Z. Other coumarins namely, Scopoletin (having IC_50_ of 41 μM for Fts-Z polymerization and 23 μM for GTPase activity) and Daphnetin (IC_50_ of 73 μM for Fts-Z polymerization and 57 μM for GTPase activity) have proven activity. In-silico studies performed on coumarins and Fts-Z indicate that it binds in the T7 loop of Fts-Z [100,101,102,103,104,105,106].

### 4.6. Curcumin

**Figure 12:**
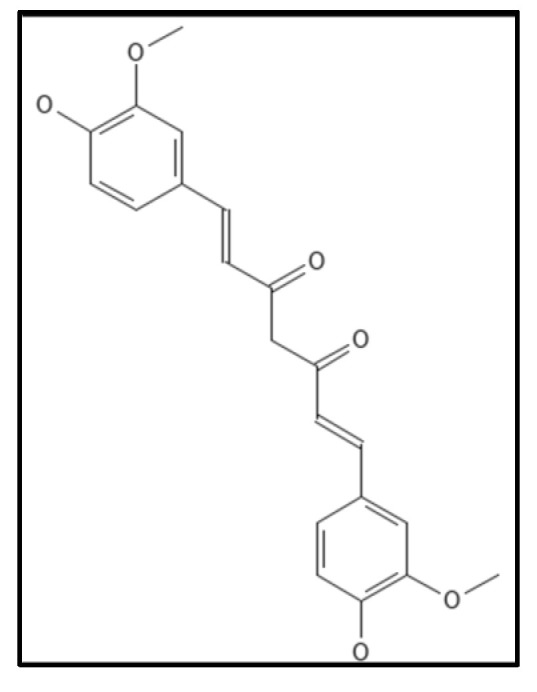
Curcumin Structure; Mol. Formula—C_21_H_20_O_6_; Mol. Wt.—368.4; Experimental details—inhibited *B. subtilis 168* (IC_50_ = 17 ± 3 µM), *E. coli* K12 MG1655 (IC_50_ = 58 ± 5 µM); dissociation constant (K_D_) = 7.3 ± 1.8 µM [108]. Source: PubChem-CID 969516.

Curcumin (IUPAC: 1,7-bis(4-hydroxy-3-methoxyphenyl)-1,6-heptadiene-3,5-dione/diferuloyl methane), (Figure 12) is a chromophore in rhizomes of the plant *Curcuma longa* (turmeric). It is a polyphenolic compound, possessing a wide spectrum of biological activities and is traditionally used in as a household remedy for curing various diseases, as spice and as a food preservative in South East Asian countries including India [88]. The presence of two ortho methoxylated phenols having conjugate linking with beta-di-ketone function makes it an appealing target for the drug industry. Rai and coworkers, revealed the inhibitory action of curcumin to polymerize Fts-Z in *B. subtilis* wherein Z-ring formation was perturbed and GTPase activity was enhanced [107]. Roy and colleagues found putative curcumin binding sites in *E. coli* Fts-Z and *B. subtilis* Fts-Z forming bonds in the GTP binding pocket through the computational docking technique [108]. Some of the research groups are working on the nano-formulation of curcumin to increase its stability in in-vitro and in-vivo setups. The major drawback of curcumin is its poor aqueous solubility and bioavailability [92].

### 4.7. Dichamanetin and Derivatives

**Figure 13:**
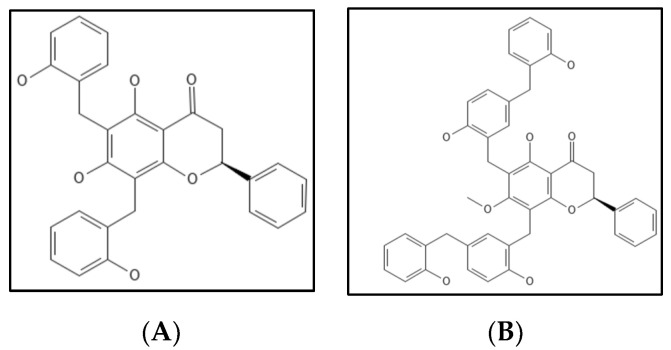
(**A**) Dichamanetin Structure; Mol. Formula—C_29_H_24_O_6_; Mol. Wt.—468.5; Experimental details—GTPase inhibition in *E.coli* (IC_50_ = 12.5 ± 0.5 µM) [109]. (**B**) 2‴-hydroxy-5″-benxzylisouvarinol-B [109]. Source: PubChem-CID 181193.

Dichamanetin (Figure 13A) is a natural polyphenolic compound obtained/isolated from Uvariachamae and 2‴-hydroxy-5″-benxzylisouvarinol-B (Figure 13B) is another natural polyphenolic compound from *Xylopia afticana* found by two independent researchers [109]. Dichamanetin and derivatives are structurally similar to Zantrins Z1, and have anti-bacterial activity against many Gram-positive microbes (*S. aureus*, *B. subtilis*, *M. smegmatis* and *E. coli*) comparable to Zantrin. Urgaonkar and colleagues examined the impact of dichamanetin on *E. coli* Fts-Z GTPase activity and found that the inhibition IC_50_ values were 12.5 µM and 8.3 µM, respectively [109].

### 4.8. Doxorubicin

**Figure 14:**
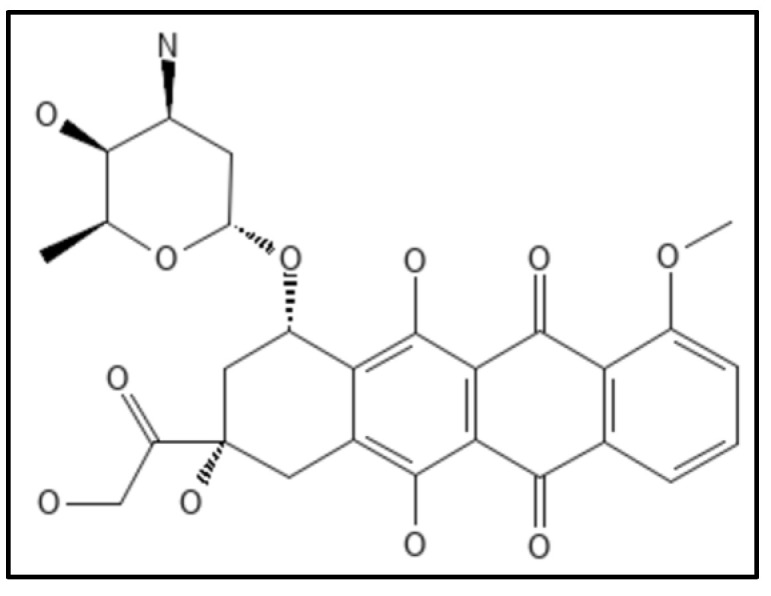
Structure of Doxorubicin; Mol. Formula—C_27_H_29_NO_11_; Mol. Wt.—543.5; Experimental details—Antibacterial activity against *E. coli* BL21, *B. subtilis* (MIC—20 µM, 10 µM respectively); Dissociation constant (*K*_D_ 120 ± 61 nM) [110]. Source: PubChem-CID 31703.

Doxorubicin, (IUPAC: (7S,9S)-7-[(2R,4S,5S,6S)-4-amino-5-hydroxy-6-methyloxan-2-yl]oxy-6,9,11-trihydroxy-9-(2-hydroxyacetyl)-4-methoxy-8,10-dihydro-7H-tetracene-5,12-dione) (Figure 14) an anthracycline antibiotic derived from the actinobacterium *Streptomyces peucetius*, has been found as a powerful Fts-Z inhibitor that inhibits *E. coli* growth by disrupting Fts-Z functions [110]. It was identified as a small molecule targeting Fts-Z and suppressing bacterial division utilizing an independent computational, biochemical, and microbiological method from a drug library authorized by the US FDA. The fluorescence-binding experiment indicated that it interacts significantly with Fts-Z without changing membrane structure or nucleoid segregation in microbes. The effects were visible on both GTPase activity and Fts-Z assembly.

### 4.9. Phenylpropanoids

**Figure 15:**
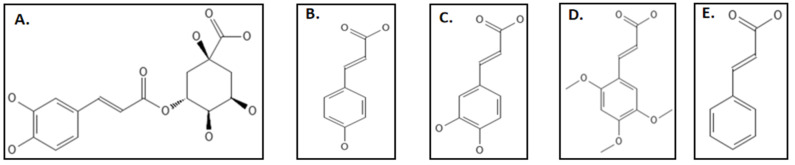
Phenylpropanoids group inhibiting Fts-Z: (**A**) Chlorogenic acid; (**B**) p-Coumaric acid; (**C**) Caffeic acid; (**D**) 2,4,5-Trimethoxycinnamic acid.; (**E**) Cinnamic acid. Source: PubChem.

Phenylpropanoids are phytochemicals produced primarily due to stress including wounding, UV irradiation, pollutants, infections, and several other environmental factors to protect them against pathogens and predators. This defensive characteristic can be attributed to their free radical hunting and antioxidant properties [111]. Studies clearly indicate the use of these phytochemicals for the production of anti-bacterial agents [111]. Hemaiswarya and coworkers studied the effect of 8 phenylpropanoids (chlorogenic acid, caffeic acid, 2,4,5-trimethoxycinnamic acid, cinnamic acid and p-coumaric acid) (Figure 15A–E), against *E. coli* Fts-Z through both in-silico and in-vitro techniques [111]. Among them chlorogenic acid which is an ester of quinic aid and caffeic acid was found to have the highest IC_50_ value of 69.55 ± 3.6 µM, caffeic acid, cinnamic acid, p-coumaric acid followed with 105.96 ± 6.3 µM, 238.91 ± 7.1 µM, 189.53 ± 3.7 µM, respectively. 2,4,5-trimethoxy cinnamic acid, 3,4-dimethoxy cinnamic acid and eugenol were the least potent (IC_50_ < 250 µM.). Light scattering experiments and circular dichroism studies supported the results. The in-silico studies indicated that the binding of phenylpropanoids happens not less than a residue in the T7 loop which is considered to be most important in the Fts-Z structure. Some other compounds which have inhibitory effects against Fts-Z were phenyl acrylamide [111,112], vanillin derivative 3a and 4u which have been tested against *S. aureus, S. pyogenes* and *M. tuberculosis* [112,113,114]

### 4.10. Plumbagin

**Figure 16:**
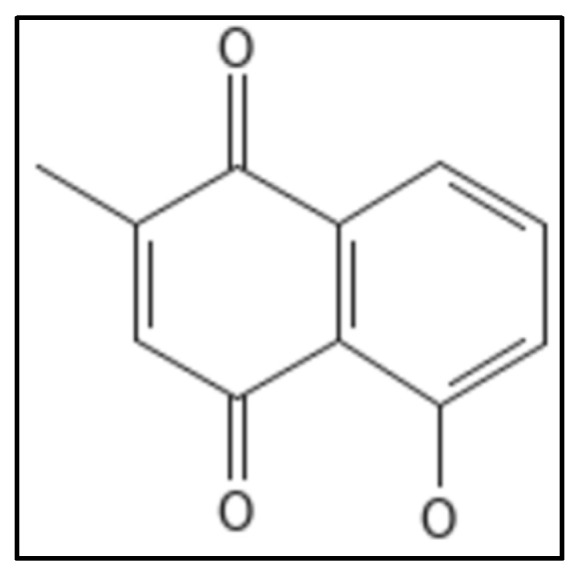
Plumbagin Structure. Mol. Formula—C_11_H_8_O_3_; Mol. Wt.—188.18; Dissociation constant (K_D_) 20.7 ± 5.6 µM [115]. Source: PubChem-10205.

Plumbagin (IUPAC: 5-hydroxy-2-methylnaphthalene-1,4-dione), (Figure 16) is a phytochemical from the roots of the *Plumbago zeylanica* plant [90]. It is a secondary naphthoquinone derivative known to exhibit a variety of biological activities such as cell proliferation in mammals [115], fungus [116] and bacterial cells [117]. The antimicrobial properties do not affect *E.coli*, *S. typhimurium* [118]. Acharya and coworkers, through their ex-vivo experiment demonstrated the binding of plumbagin with cellular microtubules in the colchicine cavity, cell viability experiments gave the IC_50_ value of 14.6 µm [119]. The structural similarity of Fts-Z with that of tubulin has caused many scientists to work on this and plumbagin was found to inhibit Fts-Z in *B. subtilis* 168 in a dose dependent manner. Further in-silico studies showed the possible binding site located at residues D199 and V307 [120].

### 4.11. Totarol

**Figure 17:**
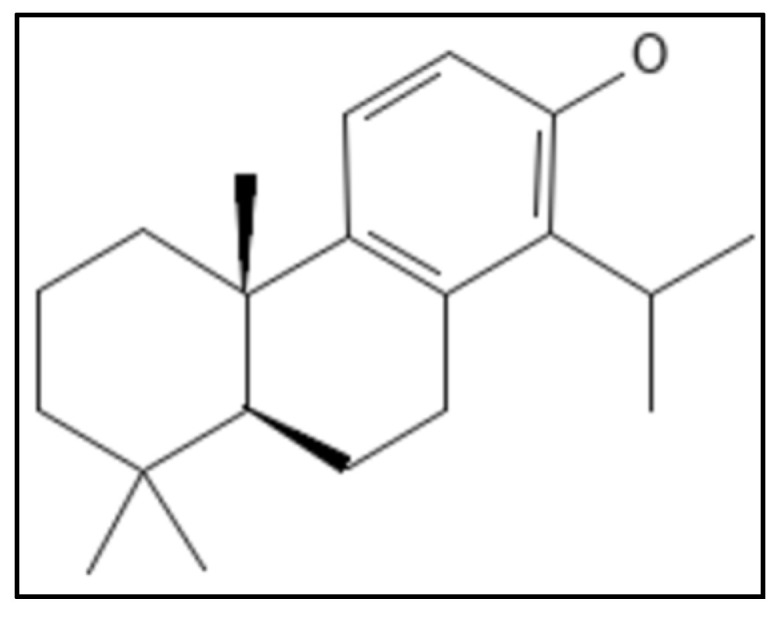
Totarol structure, Mol. Formula—C_20_H_30_O; Mol. Wt.—286.5; Experimental Details—MIC against *B. subtilis* = 2 µM, *S. aureus* = 5.4 µM, *M. tuberculosis =* 16 µg mL^−1^; Dissociation constant K_D_ = 11 ± 2.3 µM [121]. Source: PubChem-CID 92783.

Totarol, a diterpene phenolic phytocompound, (IUPAC: (4bS,8aS)-4b,8,8-trimethyl-1-propan-2-yl-5,6,7,8a,9,10-hexahydrophenanthren-2-ol) (Figure 17), is obtained from a conifer (*Podocarpus totara*) and showed activity against *M. tuberculosis* and *B. subtilis* [121,122]. Although totarol was shown to have high anti-bacterial action against a variety of Gram-positive bacteria, it had no effect on Gram-negative bacteria and no antifungal properties as well. The experiments done indicated that totarol did not disrupt the *B. subtilis* membrane or nucleoid segregation, it only affected the functioning of the Z-ring.

### 4.12. Viriditoxin

**Figure 18:**
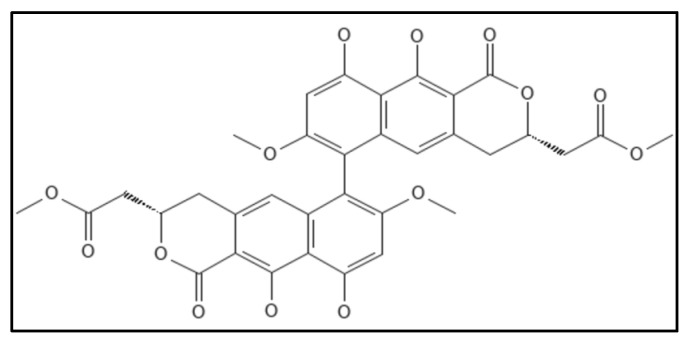
Viriditoxin Structure. Mol. Formula—C_34_H_30_O_14_; Mol. Wt.—662.6; inhibited Fts-Z polymerization (IC_50_ = 8.2 μgmL^−1^), GTPase inhibition (IC_50_ = 7.0 μgmL^−1^) [14]. Source: PubChem-53343291.

Viriditoxin (IUPAC: methyl 2-[(3S)-6-[(3S)-9,10-dihydroxy-7-methoxy-3-(2-methoxy-2-oxoethyl)-1-oxo-3,4-dihydrobenzo[g]isochromen-6-yl]-9,10-dihydroxy-7-methoxy-1-oxo-3,4-dihydrobenzo[g]isochromen-3-yl]acetate), (Figure 18) a cytotoxic compound obtained from fungus *Aspergillus* sp. (MF6890) was found to inhibit the bacterial Fts-Z polymerization by Wang and co-workers in 2003 [14]. The IC_50_ values for *E. coli* GTPase activity were 7.0 µg mL^−1^, for polymerization were 8.2 µg mL^−1^ and found to elongate *B. subtilis* cells. Another group of researchers isolated this compound from the fungus *Paecilomyces variotii* derived from jellyfish and found that viriditoxin stabilized microtubule polymers in SK-OV-3 cells and exhibited antimitotic and antimetastatic potential.

Viriditoxin is also effective against a wide range of drug-resistant Gram-positive infections, including *S. aureus* (MIC 4–8 µg mL^−1^), and *E. faecium* (MIC 2–16 µg mL^−1^). Inducing Fts-Z expression in bacterial cells may increase the MIC value, suggesting that viriditoxin targets Fts-Z in these bacterial strains. Viriditoxin can induce *B. subtilis* cells to elongate, according to morphological research [94].

### 4.13. Recent Reports

Three compounds Seltsamiayu, Galinsogisoliyu and 1H-2-Benzopyran-1-one,6,8-dihydroxy-3-(2-hydroxypropyl) from *Seltsamia galinsogisoli* Tianyuan Zhang & Yixuan Zhang, sp. nov. MB 820393, showed antimicrobial activity for *S. aureus* with MIC value of 75, 25, 32 µg mL^−1^ respectively and the in-silico docking showed a binding affinity of −109, −125, −113 kcal mol^−1^ respectively for Fts-Z protein of *S. aureus* (PDB ID—3VOB) [123]. Another research on Dysosma versipellis showed that the 4’-demethylepipodophyllotoxin compound had GTPase activity for Fts-Z of Xanthomonas oryzae *pv.* oryzae (Xoo), a plant vascular pathogen with an EC_50_ value of 38.7 mgL^−1^ yielding to filamentous cell growth as observed through transmission electron microscope and fluorescence microscopic imaging. The in-silico docking showed strong interactions at residues Asp 38, Arg 205 of Fts-Z protein of *Xoo* and the results were further strengthened with in-vivo bioassays which showed good curative and protective activities [124]. Another natural anthraquinone dye purpurin was also found to exhibit Fts-Z assembly perturbation in concentration dependent manner, by a group of researchers. In their in-vitro experiment the dissociation constant was 11 µM. It was found to bind near the nucleotide binding site and reduced GTP hydrolysis [125,126]. Naturally occurring indole alkaloids are yet another group of interest to target Fts-Z protein as studies indicate the presence of protein and enzyme inhibition potential in them. One such bis-indole containing alkaloid 34 from *Chaetomium* sp. SYP-F7950 of *Panax notoginseng*, an endophytic fungus showed anti-bacterial activity for *S. aureus, B. subtilis, E. faecium* and had MIC value of 0.12–3.6 µg mL^−1^ which was far better than vancomycin (MIC: 1.5–10 µg/mL) [127]. Another study targets Fts-Z protein through a cyanobacterial compound using in-silico and in-vitro methods and Alpha dimorphecolic acid was shown to have good anti-bacterial activity with a MIC of 512 µg mL^−1^ [128]. Some other synthetic molecules which have Fts-Z inhibition caliber are in Table 2.

### 4.14. Natural Compounds over Synthetic Drugs: A Comparison

Natural products (NPs) have been an inexhaustible source of therapeutic drugs since ancient times and numerous effective molecules have been synthesized to resemble the activity of natural compounds [139]. Almost all natural compounds have specialized biological functions for which they bind to receptors. These have pros and cons over the synthetic molecules. When compared to synthetic compound libraries, they typically have an increased molecular mass, more SP^3^ carbon and oxygen atoms but fewer nitrogen and halogen atoms, more H-bond acceptors and donors, lower calculated octanol–water partition coefficients (cLogP values, indicating higher hydrophilicity), and lesser molecular flexibility. These distinctions can be beneficial; for example, the increased rigidity of NPs can be beneficial in drug development for protein–protein interactions [140,141]. The improved methods of extracting, screening and profiling have given researchers powerful tools in the hands of researchers to explore NPs in more advanced ways.

Antibiotics are being used to cure human, veterinary and plant diseases and increase the growth and life expectancy but research suggest the long term usage of antibiotics increases negative effects on the treated organisms [142]. There are more than 250 chemical substances registered to be used in different countries for humans and farm animals [143]. Antibiotics given to animals in food or water to increase their growth and to protect them from infections developing in them due to unhealthy living conditions. When they are not completely metabolized in the body, they are discharged by the process of adsorption/desorption into the environment as contaminants. They then enter the food chain via manure application or grazing animals and accumulate in edible plant parts [144]. Some of them also ingested through meat into the human body [145]. Certain studies show deleterious effects, delayed germination and post germinative development in plants due to antibiotics [131]. The presence of antibiotics in soil, groundwater, poultry meat and other plant and animal-based products and the side effects caused by antibiotics in environment are staggering which has caused the scientific community and agencies to reassess their use [146,147,148,149,150,151,152,153]. Thus, more efforts are being made to employ natural products for fighting pathogens.

## 5. Discussion

Fts-Z is an important cytoskeleton protein that is currently being explored and targeted by several natural and synthetic compounds in a number of pathogenic bacterial species. Many bacterial cytoskeletal proteins such as MreB, ParM, and MamK, Min C and Zap proteins similar to Fts-Z or with any new activities are also being investigated as potential antibiotic discovery candidates [154]. The key cell division proteins targets are highly conserved in most of the bacteria [65], however, there are a number of other proteins unique to each genus are very promising [155]. Hence, inhibiting these could be an alternative strategy for the discovery for characterization of novel antimicrobial drug targets.

Most antibiotics from the present generation primarily target either cell wall biosynthesis or protein/nucleic acid synthesis [156]. Exploring and analyzing comprehensive data on Fts-Z structure is also done in the recent years revealing its functional mechanisms [157]. Many successful studies has been conducted with natural inhibitors to target bacteria such as *S. aureus*, *E. coli*, *B. subtilis*, *VRE*, *M. tuberculosis*. Though, several studies have been conducted with both natural and synthetic inhibitors, in this review we have covered the significant studies on the natural inhibitor of Fts-Z performed in recent years

Primarily, the natural compounds or their synthetic derivatives which exhibit a potential binding affinity with Fts-Z are being studied as a drug candidates [72,73,74,75]. Amongst the presently known inhibitors, viriditoxin showed a broad spectrum of activity by inhibiting GTPase and polymerizing Fts-Z [123]. Other compounds that have also been studied are sanguinarines [28,89], totarols [121,122], dichamanetins [107], 2‴-hydroxy-5″-benzylisouvarinol-B [107], zantrins [129], curcumin [105], and cinnamaldehyde [94]. Some of the natural inhibitors show a strong activity through either GTPase inhibition or polymerizing activity.

Such activities were tested for both Gram-positive and Gram-negative bacteria. Some studies were performed using in-vitro techniques while others used in-silico and in-vivo experiment. After such extensive research on this target, compounds like TXA 709, a prodrug was studied in the phase I trials [59], thus increases the interest of scientists to find a drug to target this crucial protein necessary for divisome machinery. Additionally, it has recently been demonstrated that TXA707 acts synergistically with the third-generation cephalosporin cefdinir against several multidrug-resistant *S. aureus* [158]. Thus, medicinal chemists are working intensively on combinatorial therapies to increase the efficiency of drugs along with broadening the drug spectrum against tuberculosis, HIV and cancer and also to include Gram-negative microorganisms.

Another benzamide derivative PC190723 has selectively potent anti-staphylococcal activity (MIC < 2.81 µg mL^−1^) and has sown a seed for further trials in this direction. Further drugs are being worked on having similar structural scaffolding as of PC190723 [133].

In this regard, investigations on the combination of PC190723 prodrug (TXY436) with an efflux-pump inhibitor (i.e., PAβN) revealed that the presence of sub-inhibitory concentrations of PAβN confers TXY436 with activity against Gram-negative strains (namely, *E. coli, P. aeruginosa*, and *A. baumannii*). It is envisaged that these impressive results, along with the previously reported Fts-Z inhibitors [128], will pave the way for the creation of a new class of anti-bacterial chemical entities that will inhibit bacterial cytokinesis and have substantial therapeutic utility.

The natural inhibitors of Fts-Z showing comparable inhibition activities need to be taken further for in-vivo and clinical trials as they would certainly be comparatively less toxic than the synthetic compounds/inhibitors.

Research suggests the viability of Fts-Z as promising anti-bacterial target [13,49,50,51]. It was suggested that an early generation of resistance might occur in new drugs, as happens in other single-target inhibitors which although this can be prevented/avoided by tactical and amenable use of combination therapy. Another point of concern is that most Fts-Z inhibitors to date are broad-spectrum anti-bacterial which damage the host flora and fauna and thus should be put off if feasible [35,100]. In addition to this, these broad spectrum antibiotics aid the bacterial stress response, elevating the chance of bypassing the host’s innate immune response [51,52,53]. There is also need to check if bacterial cells regain normal division pattern once the inhibitor is removed using the fragmentation of the filament technique [54].

Another important point of concern is that Fts-Z is homologous to tubulin, although without any homology in amino acid sequence yet there is a need for critical evaluation of its impact on the function of tubulin in hosts [55,56]. Some studies also showed that for successful inhibition of beta lactams, well-functioning cell division machinery mandatory [57]. We need to keep the above mentioned points and related works in mind while developing Fts-Z inhibitors for the clinical trials.

## 6. Conclusions

Targeting bacterial divisome protein Fts-Z is emerging as a promising strategy for the identification of new antibiotic compounds as well as to deal with AMR. Several studies reported that Fts-Z inhibitors could provide scaffolds and pharmacophores for the further development of novel anti-bacterial agents. In the pursuit of novel anti-bacterial compounds, targeting natural products are gaining significant attention as synthetic compounds are leading to development of several antibiotic resistant strains of bacteria. Natural compounds are always the safest substitute in terms of toxicity, environmental persistence and effects on non-target organisms. A few potential compounds discussed in this review are believed to be promising, however, how these inhibitors performed in the future research; will set the fine course for the anti-bacterial drug development using the natural compounds. 

## Figures and Tables

**Table 1:** Summary of essential cell division protein (source: Seidel, Reyes et al., Geissler et al., Lock and Harry [47,50,51,52]).

Protein	Function	Interacting Protein
Fts-Z	Cytoskeleton protein, Self-polymerizing GTPase which forms Z-ring/proto ring and recruitment of other proteins	Fts-A, Zip-A, Fts-K
Fts-A	ATP binding protein which anchors and stabilizes Fts-Z filaments	Fts-N, Fts-Z
Zip-A	Provides membrane anchorage for Fts-Z by bunding the filaments	Fts-Z
Fts-K	DNA segregation at C terminal, stabilizing component and linker between early and late stages of division.	Fts-A, Fts-I, Fts-L, Fts-Q, Fts-Z
Fts-Q	Periplasmic functional domain, Peptidoglycan synthesis, Complex formation with Fts-B and Fts-L	Fts-B, Fts-I, Fts-L, Fts-N, Fts-W
Fts-L	Complex formation with Fts-B and Fts-Q, Peptidoglycan synthesis	Fts-B, Fts-I, Fts-K, Fts-Q
Fts-B	Complex formation with Fts-L and Fts-Q, Peptidoglycan synthesis	Fts-L, Fts-Q
Fts-W	Translocation of lipid precursors across membrane, Peptidoglycan synthesis	Fts-I, Fts-L, Fts-N, Fts-Q, Fts-Z
Fts-I	Transpeptidase, Cross links peptidoglycan strains	Fts-N, Fts-Q, Fts-W
Fts-N	Triggers septation during peptidoglycan synthesis, Periplasmic functional domain	Fts-A, Fts-I, Fts-Q, Fts-W

**Table 2:** Synthetic and semi-synthetic inhibitors over natural inhibitors.

Compound	Target Organism	Action Mechanism	Assay Used	MIC/IC_50_	Ref.
Zantrin	Broad range	Z1, Z2, Z4 destabilize Fts-Z assembly; Z5 hyper stabilize Fts-Z assembly.	Real time enzyme coupled fluorescent GTPase assay.	*E. coli* = 4–25 µM *M. tuberculosis* = 30–70 µM	[129]
Amikacin	*E. coli*	Z-ring perturbation	Cell elongation	4 µg mL^−1^	[130]
A-189	*E. coli*, *S. aureus*	GTPase inhibition and Z-ring assembly inhibition	Anucleate cell blue assay.	16 µg mL^−1^	[131]
GTP analogue	*E. coli*, *S. aureus*	GTPase inhibition	Spectrophotometric coupled enzymatic assay	MeOGTP-IC_50_ = 15 μM	[132]
PC190723	*B. subtilis*, *S. aureus (MRSA)*	Binds H7 loop affecting GTPase activity causing Z-ring mis localization	Whole cell-based assay leading to filamentous phenotype	0.5 µg mL^−1^	[133]
SRI-3072	*M. tuberculosis*	Inhibition of Fts-Z polymerization and GTPase activity	Antimicrobial assay	19 µM	[134]
Taxanes	*M. tuberculosis*	Stabilizes Fts-Z against depolymerization	Real-time PCR based assay, Cell filamentation	1.25−2.5 μM	[76]
2-carbamoyl pteridine	*M. tuberculosis*	GTPase activity inhibition and Fts-Z polymerization	GTPase activity and Fts-Z polymerization through in vitro technique	2 µg mL^−1^	[135]
534F6 derivatives	*E. coli*	Cell division inhibition	Microorganisms lacking MDR pumps were used for screening	>80 μM	[136]
Bis indole methane (2,2′-bisindole)	*M. tuberculosis*, *M. segmentis*, *H37Rv strain*	Cell division inhibition	GTPase activity, Cell proliferation, Antimycobacterial property	62.5 µg mL^−1^	[137]
5-Methyl-11-((3-(3-(4-methylpyridine))-propyl benzo[d] thiazol-2(3H)-ylidene) methyl) benzofuro[3,2-b] quinolin-5-ium iodide)	*B. subtilis*, *S. aureus*, *E. coli*	GTPase activity, Polymerization of Fts-Z	Cell based antibiotic screening assay, Biochemical assay, Light scattering assay	0.5, 2, 4 µg mL^−1^ respectively	[138]

## Data Availability

Not applicable.

## Abbreviations

AMR	Anti Microbial Resistance
ATCC	American Type Culture Collection
ESKAPE	*Enterococcus faecium*, *Staphylococcus aureus*, *Klebsiella pneumoniae*, *Acinetobacter baumannii*, *Pseudomonas aeruginosa*, and *Enterobacter* species
GTP	Guanosine-5’-triphosphate
MRSA	Methicillin-resistant *Staphylococcus aureus*
MGEs	Mobile Genetic Elements
NDM	New Delhi Metallo beta-lactamase-I
MDR	Multi Drug Resistance
PDR	Pan drug-resistant
VRSA	Vancomycin-resistant *Staphylococcus aureus*
WHO	World Health Organization
NP	Natural Products
VRE	Vancomycin-Resistant *Enterococci*
